# Association of *TERC* and *OBFC1* Haplotypes with Mean Leukocyte Telomere Length and Risk for Coronary Heart Disease 

**DOI:** 10.1371/journal.pone.0083122

**Published:** 2013-12-12

**Authors:** Cécilia G. Maubaret, Klelia D. Salpea, Casey E. Romanoski, Lasse Folkersen, Jackie A. Cooper, Coralea Stephanou, Ka Wah Li, Jutta Palmen, Anders Hamsten, Andrew Neil, Jeffrey W. Stephens, Aldons J. Lusis, Per Eriksson, Philippa J. Talmud, Steve E. Humphries, the Simon Broome Research Group , the EARSII consortium

**Affiliations:** 1 Cardiovascular Genetics, BHF Laboratories,University College London (UCL), London, United Kingdom; 2 ISPED, Université Bordeaux Ségalen/INSERM u.897, Bordeaux, France; 3 Institute of Molecular Biology & Genetics, Biomedical Sciences Research Center "Alexander Fleming", Athens, Greece; 4 Department of Human Genetics, University of California Los Angeles, Los Angeles, California, United States of America; 5 Atherosclerosis Research Unit, Center for Molecular Medicine, Department of Medicine, Karolinska Institutet, Stockholm, Sweden; 6 Atherosclerosis Research Unit, Department of Medicine Solna, Karolinska Institutet, Stockholm, Sweden; 7 Division Public Health & Primary Health Care, University of Oxford, Oxford, United Kingdom; 8 Diabetes Research Group, School of Medicine, Swansea University, Swansea, United Kingdom; Medical University Hamburg, University Heart Center, Germany

## Abstract

**Objective:**

To replicate the associations of leukocyte telomere length (LTL) with variants at four loci and to investigate their associations with coronary heart disease (CHD) and type II diabetes (T2D), in order to examine possible causal effects of telomere maintenance machinery on disease aetiology.

**Methods:**

Four SNPs at three loci *BICD1* (rs2630578 GγC), 18q12.2 (rs2162440 GγT), and *OBFC1* (rs10786775 CγG, rs11591710 AγC) were genotyped in four studies comprised of 2353 subjects out of which 1148 had CHD and 566 T2D. Three SNPs (rs12696304 CγG, rs10936601G>T and rs16847897 GγC) at the *TERC* locus were genotyped in these four studies, in addition to an offspring study of 765 healthy students. For all samples, LTL had been measured using a real-time PCR-based method.

**Results:**

Only one SNP was associated with a significant effect on LTL, with the minor allele G of *OBFC1* rs10786775 SNP being associated with longer LTL (β=0.029, P=0.04). No SNPs were significantly associated with CHD or T2D. For *OBFC1* the haplotype carrying both rare alleles (rs10786775G and rs11591710C, haplotype frequency 0.089) was associated with lower CHD prevalence (OR: 0.77; 95% CI: 0.61–0.97; P= 0.03). The *TERC* haplotype GTC (rs12696304G, rs10936601T and rs16847897C, haplotype frequency 0.210) was associated with lower risk for both CHD (OR: 0.86; 95% CI: 0.75-0.99; P=0.04) and T2D (OR: 0.74; 95% CI: 0.61–0.91; P= 0.004), with no effect on LTL. Only the last association remained after adjusting for multiple testing.

**Conclusion:**

Of reported associations, only that between the *OBFC1* rs10786775 SNP and LTL was confirmed, although our study has a limited power to detect modest effects. A 2-SNP *OBFC1* haplotype was associated with higher risk of CHD, and a 3-SNP *TERC* haplotype was associated with both higher risk of CHD and T2D. Further work is required to confirm these results and explore the mechanisms of these effects.

## Introduction

Telomeres are made up of TTAGGG sequences repeated across four to 15 kilobases at the end of each chromosome. These specialised ribonucleoprotein structures, which prevent chromosome degradation and abnormal DNA repair, are essential for preserving the integrity of genetic information [1,2]. DNA replication is incomplete at the 3’end of the linear chromosome, owing to the so-called “end-replication” problem. Therefore, in most adult differentiated cells, telomeres shorten progressively with each cell division until they reach a critical length (the Hayflick limit), where the cell cycle is interrupted and the cells enter senescence [3,4] . To prolong their growth capacity, highly dividing cells, such as stem cells, maintain high activity of the telomerase complex, composed of the reverse transcriptase *TERT* and the RNA template *TERC*, responsible for the replenishment of shortened telomeres [5,6].

In cross-sectional studies, shorter mean leukocyte telomere length (LTL) was reported in atherosclerotic [7] and myocardial infarction (MI) patients [8,9] compared to healthy subjects. Similarly, compared to controls, a shorter mean LTL was reported in type II diabetic (T2D) patients [10,11]. LTL shortening was proposed to reflect the cumulative burden of inflammation and oxidative stress throughout the lifespan of an individual, rendering LTL a suitable marker for aging [12]. Peripheral blood LTL has been shown to correlate with aortic cells telomere length (TL), and thus mean LTL could be used as a surrogate marker of the aortic cells mean telomere length [13]. However, the causal effect of short telomere on heart disease development remains controversial. 

Twin studies and intra-familial correlation analysis showed that TL is highly heritable, with up to 80% of LTL variation being genetically determined [14,15]. The first SNP to be associated with LTL (rs2630778) was located in the first intron of the *BICD1* gene [16]. In 2009, a *locus* in the chromosome region 18q12.2 was associated with short TL in European population [17]. Further analysis in a combined sample of six cohorts identified association between TL and a locus on the chromosome region 3q26, which includes the *TERC* gene, although the*BICD1*and the 18q12.2 association with LTL was not replicated in this larger sample [18]. The association between LTL and *TERC* SNPs was confirmed in different studies [19,20]. The product of the *TERT* gene is a critical key component of the telomerase complex, and some but not all studies showed association between SNPs at this locus and LTL [21–23]. Recently, Levy et al. [24] identified an association between SNPs in the region of the *OBFC1* gene with LTL and confirmed the association between TL and SNPs in the vicinity of the *TERC* gene. However, all studies to date showed a modest SNP effect size on LTL especially *TERC-*linked SNPs, meaning that replication studies are required to obtain robust results. 

In the present study, we attempted to replicate the previously reported associations between LTL and SNPs in the *BICD1* and *OBFC1* genes, at the 18q12 locus and near the *TERC* gene. Additionally, using CHD and T2D case-control designs, we investigated whether these associations could be seen in CHD and T2D patients, in order to test the potential modifying effect of health status on the association strength. Finally, we investigated the direct effect of the SNPs on the risk of CHD and T2D, since this is a way to interrogate the potential causality of telomere length maintenance system on disease pathology.

## Results

All analyses were restricted to individuals of European ancestery. The mean values of the general characteristics of the subjects in each study are presented in Table 1. A shorter mean LTL was observed in older compared to younger subjects as well as in cases compared to controls, as previously reported [9,11,25].

**Table 1:** Characteristics of the studies subjects.

	HIFMECH		CABG	SB-FH		UDACS		EARSII		
	controls	MI cases	CHD cases	- CHD	+ CHD	- CHD	+ CHD		controls	“MI” cases	
Number	559	520	341	222	145	424	142		396	369	
% women	0	0	20.8	56.8	33.8*	43.2 (183)	31.7 (45)*		0	0	
Age (years)	51.1 (5.5)	51.9 (5.4)	64.9 (9.2)	44.3 (13.4)	56.1 (10.3)*	65.8 (11.3)	69.3 (9.7)*		22.7 (0.1)	22.7 (0.1)	
BMI (kg/m^2^)	26.1 (3.2)	27.1 (3.3)*	28.2 (4.5)	23.8 (4.0)	25.1 (3.4)*	29.3 (5.6)	29.6 (5.4)		23.3 (0.1)	23.4 (0.1)	
CRP (mg/l)	1.23 (1.40)	2.22 (2.55)*	2.29 (2.77)	1.16 (1.34)	1.42 (1.59)	1.70 (1.45)	1.82 (1.60)		0.43 (0.50)	0.48 (0.57)	
Cholesterol (mmol/l)	5.52 (0.97)	5.40 (1.18)	4.71 (1.03)	6.91 (1.30)	6.35 (1.31)*	5.19 (1.07)	4.73 (1.12)*		4.30 (0.04)	4.53 (0.04)*	
Glucose (mmol/l)			6.19 (2.05)	4.82 (0.59)	5.07 (0.59)*	9.96 (4.31)	9.65 (4.24)		5.17 (0.02)	5.16 (0.02)	
Insulin (mU/l)	37.8 (24.3)	49.3 (34.5)*				-	-		10.6 (0.2)	10.1 (0.2)	
Unadjusted LTL (kb)	8.04 (4.51)	7.90 (4.05)	6.85 (4.23)	9.82 (4.88)	9.04 (4.74)*	6.98 (3.50)	6.80 (3.50)		9.09 (6.61)	8.58 (6.71)	

For age and cholesterol results are mean (standard deviation). For BMI, CRP, Glucose, insulin and LTL results are geometric mean (approximate standard deviation). EARS “MI” cases are actually sons of MI patients.

BMI, body mass index; LTL, leukocyte telomere length.

*P* values are from two-sample t-test; LTL were adjusted for age, gender, center as appropriate using multiple regression. For the UDACS study, analysis was limited to the Caucasian population.

^*^ indicates that the difference between cases and controls was significant with p<0.05.

### SNPs genotype distribution and linkage disequilibrium

Table 2 shows the seven SNPs selected for genotyping along with previously reported effect sizes and analytical models used. In the HapMap database, the linkage disequilibrium as assessed by r^2^ was 0.35 between rs12696304 and rs16847897 (*TERC*), 1.0 between rs12696304 and rs10936601 (*TERC*) and 0.64 between rs10786775 and rs11591710 (*OBFC1*) for the CEU population. Overall, genotypes were obtained for 92% of the samples (ranging from 87% for rs2162440 to 93% for rs12696304). In our control samples, the genotype distribution for all SNPs was in Hardy-Weinberg equilibrium (p>0.05). 

**Table 2:** List of the SNPs genotyped in this study with previously reported β coefficient.

SNP	Locus	β coefficient (SE)	P	Model	Allele associated with shorter LTL	References
rs2630578	*BICD1*	-0.604 (0.204)	1.9 x 10^-5^	Dominant	C	16,18
rs2162440	18q12.2	-0.106 (0.022)	2.6 x 10^-6^	Additive	G	17,18
rs16847897	*TERC*	-0.030 (0.008)	9.33 x 10^-5^	Additive	C	18
rs12696304	*TERC*	-0.034 (0.008)	1.03 x 10^-5^	Additive	G	18,22
rs10936601	*TERC*	0,073 (0,034)	0.034	Additive	G	our unpublished GWAS (AETL) 2010
rs10786775	*OBFC1*	-0.120 (0.020)	6.13 x 10^-8^	Additive	C	22
rs11591710	*OBFC1*	-0.110 (0.020)	7.87 x 10^-8^	Additive	A	22

SE, standard error. B coefficient was directly copied from the publication and direction of the association will depend on the calculation.

The attention of the reader is attracted to the fact that all data were obtained using leucocytes telomeres length measurement for GWAS screen except our unpublished GWAS-AETL which was performed on aortic endothelial telomere length.

### SNPs genotype and association with LTL

**Figure 1:**
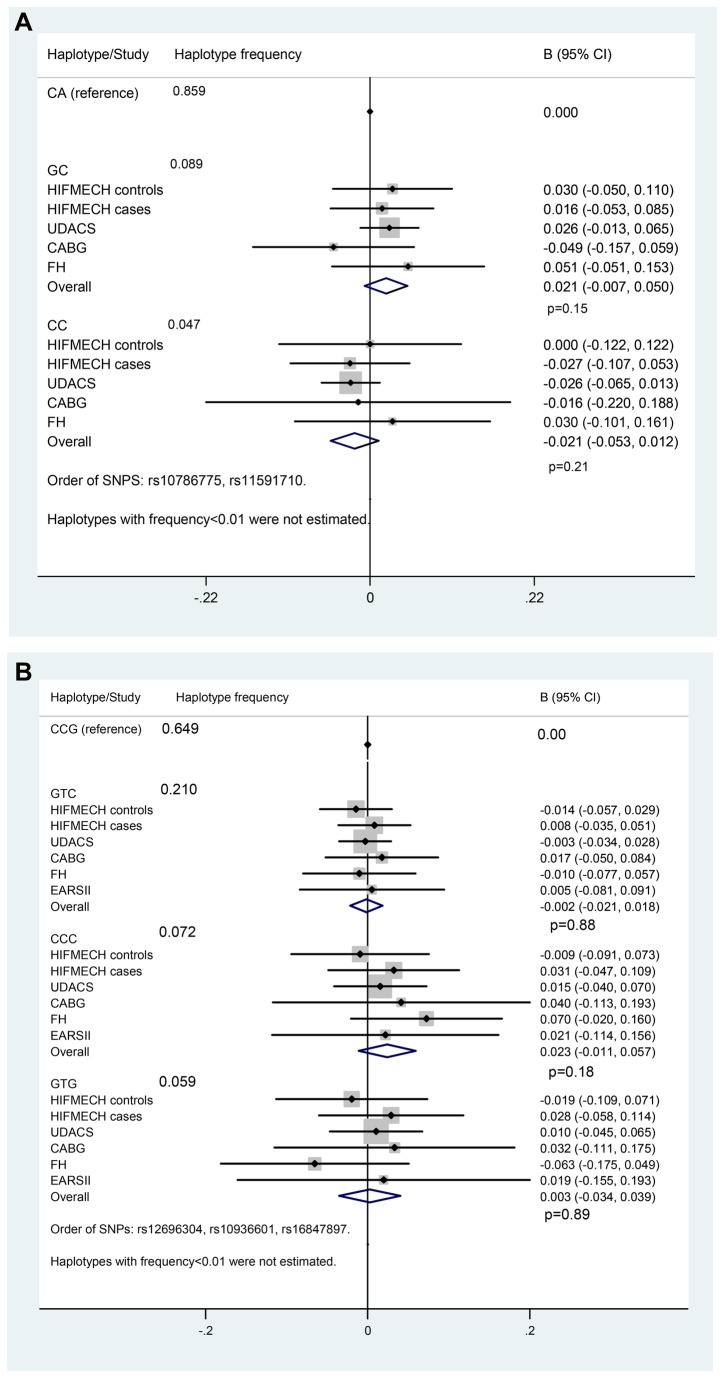
LTL by *OBFC1* haplotype, (A). LTL by TERC haplotype, (B).

The allele frequency is each study is presented in Table S1 in File S1. The association of the adjusted LTL with each SNP genotype in each study is presented in Table S2 in File S1 and the meta-analysis is presented in Table 3. As generally expected for SNPs with a modest effect in small-size samples, the differences between mean LTL by genotypes were not statistically significant in the individual study samples. In the four studies (n=2353) meta-analysis, a significant association between the rare allele G of the *OBFC1* rs10786775 SNP and longer LTL (β=0.029, *P*=0.04) was observed. Figure 1A illustrates the effect of the 2-SNP *OBFC1* haplotype on LTL. Although it did not reach conventional levels of statistical significance, the association between the haplotype GC and longer LTL was consistent in four out of five study samples and directionally the same as previously reported [24].The 3-SNP *TERC* haplotype effect is presented in Figure 1B. No significant association with LTL was detected.

**Table 3:** Test for association of the SNPs with LTL (leukocyte telomere length) in combined studies.

SNP	Minor allele	Minor allele frequency	β	P
rs2630578 (*BICD1*)	C	0.162	0.010	0.38
rs2162440 (18q12.2)	T	0.203	-0.018	0.46
rs16847897 (*TERC*)	C	0.284	0.003	0.70
rs12696304 (*TERC*)	G	0.280	-0.003	0.72
rs10936601 (*TERC*)	T	0.275	-0.003	0.73
rs10786775 (*OBFC1*)	**G**	**0.095**	**0.029**	**0.04**
rs11591710 (*OBFC1*)	C	0.135	0.013	0.27

The β coefficient is given for each copy of the minor allele.

### SNPs genotype and CHD risk

**Figure 2:**
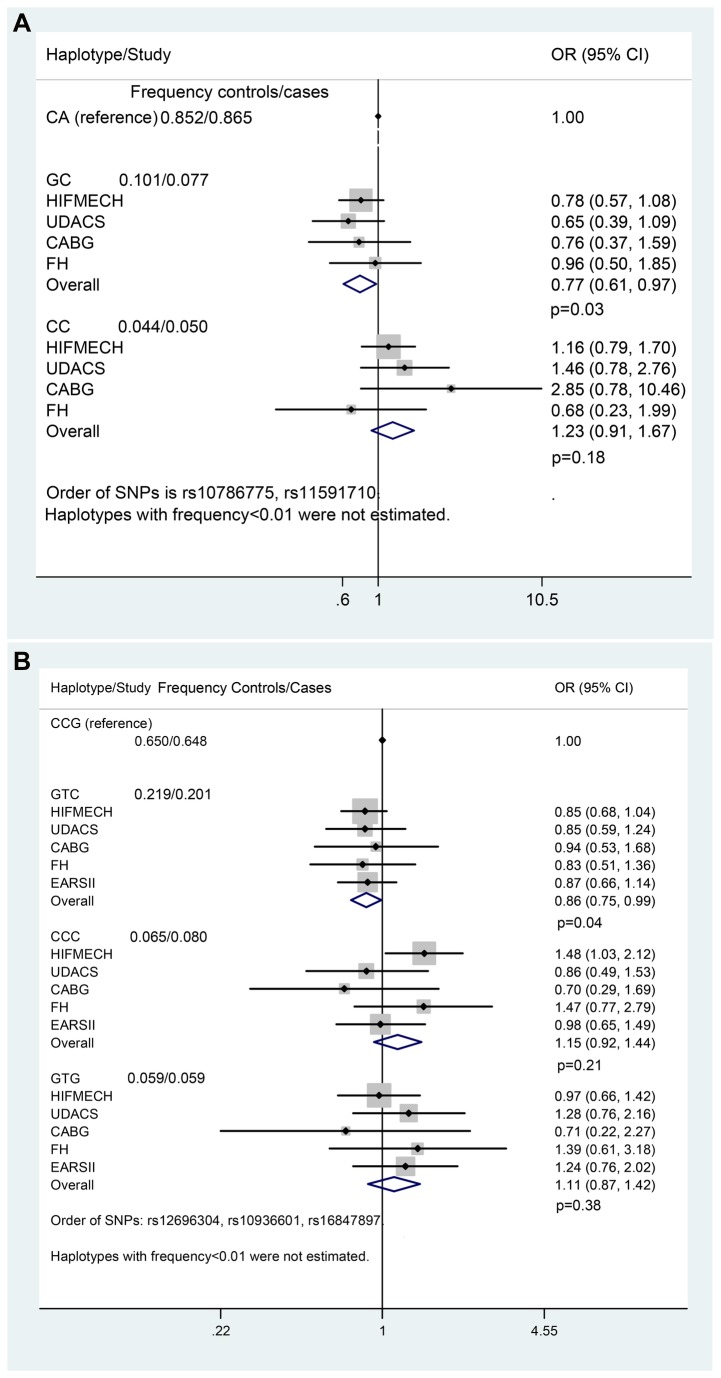
Forest plot of the effect of *OBFC1* haplotype on CHD risk, after adjustment for LTL, (A). Forest plot of the effect of *TERC* haplotype on CHD risk, after adjustment for LTL, (B).

Table 4 shows the age, gender, centre, physical activity (HIFMECH) adjusted odds ratio (OR) for CHD for each SNP in individual studies. In the HIFMECH study, carriers of the rare allele T of rs10936601 (*TERC*) showed significantly lower risk (19%) for CHD compared to non-carriers (OR: 0.81; 95% CI: 0.66 -0.99, P=0.04). The trend for a lower OR for the rare allele was consistent in CABG (compared to HIFMECH UK controls), in SB-FH and in EARSII, which is an offspring study. Nevertheless, the association was not observed for rs12696304 SNP in strong LD with rs10936601. The *OBFC1* haplotype GC (haplotype frequency 0.089) was associated with a lower prevalence (23%) for CHD (OR: 0.77; 95% CI: 0.61-0.97; *P*= 0.03) (Fig. 2A). Using *TERC* haplotype meta-analysis (Figure S1 in File S1), the *TERC* haplotype GTC (haplotype frequency 0.210) was found to be significantly associated with a lower CHD prevalence (OR: 0.87; 95% CI: 0.76 -1.00, P= 0.05) in five studies (n = 3118, 1601 controls and 1517 CHD cases). The association did not change after adjustment for LTL (Figure 2B).

**Table 4:** Test for association of the SNPs with CHD.

SNP	UDACS	HIFMECH	CABG	SB-FH	EARS	All CHD studies
rs2630578	0.84 (0.57-1.23)	0.90 (0.70-1.16)	0.74 (0.40-1.37)	0.86 (0.56-1.32)	NA	0.89 (0.78-1.02)
(*BICD1*)	P=0.37	P=0.41	P=0.34	P=0.49		P=0.12
rs2162440	1.35 (0.97-1.86)	0.96 (0.75-1.21)	0.79 (0.43-1.43)	0.93 (0.62-1.39)	NA	1.03(0.87-1.22)
(18q12.2)	P=0.07	P=0.70	P=0.44	P=0.73		P=0.74
rs16847897	0.85 (0.62-1.16)	%1.1 (0.83-1.24) P=0.90	0.82 (0.51-2.32)	1.01 (0.7-1.46)	0.94 (0.74-1.21)	0.96 (0.84-1.09)
(*TERC*)	P=0.30	%1.2	P=0.42	P=0.96	P=0.62	P=0.50
rs12696304	0.98 (0.72-1.32)	0.86 (0.71-1.05)	0.83 (0.52-1.33)	1.01 (0.69-1.49)	0.89 (0.70-1.13)	0.90 (0.80-1.02)
(*TERC*)	P=0.88	P=0.14	P=0.45	P=0.96	P=0.34	P=0.09
rs10936601	1.06 (0.78-1.43)	**0.81 (0.66-0.99)**	0.77 (0.48-1.22)	0.95 (0.65-1.40)	0.94 (0.74-1.21)	0.89 (0.79-1.01)
(*TERC*)	P=0.73	**P=0.04**	P=0.26	P=0.80	P=0.62	P=0.08
rs10786775	0.64 (0.38-1.06)	0.84 (0.61-1.16)	0.75 (0.38-1.48)	1.00 (0.57-1.77)	NA	0.81 (0.64-1.02)
(*OBFC1*)	P=0.08	P=0.30	P=0.40	P=0.996		P=0.07
rs11591710	0.84 (0.56-1.27)	0.90 (0.69-1.17)	1.13 (0.59-2.16)	0.89 (0.54-1.49)	NA	0.90 (0.74-1.10)
(*OBFC1*)	P=0.40	P=0.42	P=0.72	P=0.66		P=0.30

Non adjusted for LTL.

Odds ratio and 95% confidence interval are presented, NA; not available;

### SNPs genotype and T2D risk

Table 5 presents the test for associations of each SNP in single SNP analysis with T2D comparing UDACS cases with HIFMECH controls. Analyses were adjusted for age, gender, centre, and exercise. None of the individual SNPs were associated with T2D. A three-SNPs *TERC* haplotype analysis showed a significantly lower OR for haplotype GTC compared to the reference haplotype CAC (OR: 0.74; 95% CI: 0.61–0.91; *P*=0.004) (Table 6) in the UDACS T2D cases (n = 566) compared to the HIFMECH controls (n = 559). Both haplotype and single SNP analyses, reported a non-significant effect of*OBFC1* genotypes on the risk for T2D (data not shown). 

**Table 5:** Test for association of the SNPs with T2D UDACS (compared to HIFMECH controls).

SNP	UDACS
rs2630578(*BICD1*)	%1.3 (0.82-1.29)P=0.79
rs2162440(18q12.2)	1.07 (0.87-1.32)P=0.51
rs16847897(*TERC*)	0.85 (0.71-1.02)P=0.07
rs12696304(*TERC*)	0.88 (0.74-1.06)P=0.17
rs10936601(*TERC*)	0.88 (0.74-1.05)P=0.17
rs10786775(*OBFC1*)	%1.1 (0.77-1.34)P=0.92
rs11591710(*OBFC1*)	0.94 (0.74-1.19)P=0.59

**Table 6:** Type II diabetes by *TERC* haplotype (UDACS compared to HIFMECH controls).

Haplotype	Freq in controls	Freq in T2D	OR (95% CI)	OR (95% CI) adjusted for LTL
CCG	0.613	0.646	1.00	1.00
GTC	0.259	0.199	**0.74 (0.61-0.91) P=0.004**	0.83 (0.69-1.01) P=0.07
GTG	0.058	0.077	1.26 (0.89-1.80) P=0.20	1.36 (0.98-1.89) P=0.06
CCC	0.054	0.064	1.12 (0.78-1.60) P=0.55	1.00 (0.72-1.39) P=1.00
Global p value			**P=0.007**	**P<0.0001**

Order of SNPs is rs12696304 rs10936601 and rs16847897

### Gene expression

**Figure 3:**
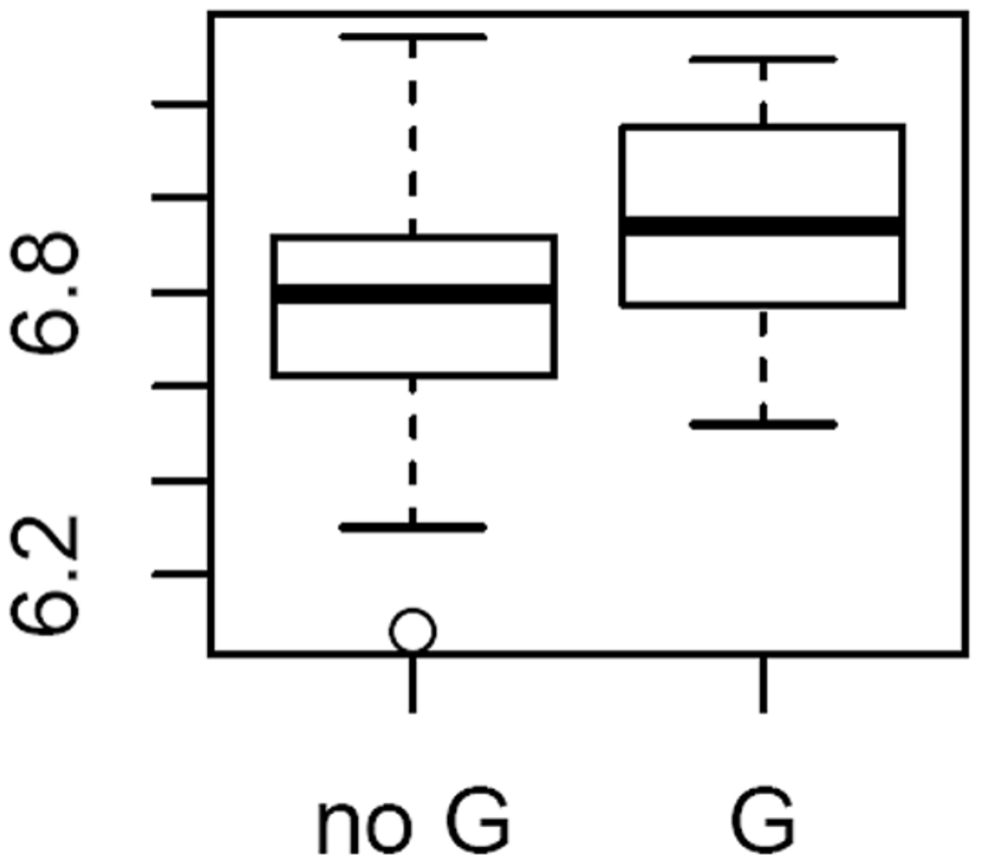
rs10786775 genotype associates with *OBFC1* expression in adventitial aortic tissue. *OBFC1* expression level by rs10786775 genotype. Sample distribution was 115 no G // 18 G (17GC+1 GG).

To explore the possible mechanism of action of the variants in CHD pathology, the association between rs10786775 genotype and *OBFC1* mRNA levels was examined in aortic tissue. As shown in Figure 3, carriers of the rs10786775 SNP G allele had higher *OBFC1* expression in adventitia aortic tissue compared to homozygote carrier of the frequent allele C (P=0.01007). There were no specific *TERC* SNPs available on the Affymetrix Exon 1.0 ST (and no imputable SNPs) for expression analysis, and no strong association between rs16847897 and *TERC/ARPM1* expression. 

## Discussion

Here we confirm the association of the rare allele G of the *OBFC1* SNP rs10786775 with longer LTL, which is in accordance with Levy’s study [24]. By contrast, our replication study did not confirm the published association between the *BICD1* SNP or the chromosome 18q12.2 locus with LTL [16,17]. Therefore our results corroborate Levy’s [24] and suggest that the *BICD1* and the 18q12.2 loci do not have major influences on LTL. We did not find significant association between LTL and *TERC* SNPs in the present study. However, the absence of a significant association observed here should be interpreted with caution and could be caused by the small sample size and low study power.

Considering the large number of publications which report a shorter LTL in CHD and T2D patients compared to controls, the direct association of *TERC* and *OBFC1* haplotypes and CHD and T2D was investigated. Alongside work by Zee et al. [26,27], our study is one of the first to examine the direct effect of telomere maintenance genes variants on CHD and T2D risk. In our dataset, the *TERC* haplotype GTC was significantly associated with a lower OR for CHD (OR: 0.86; 95% CI: 0.75 - 0.99; *P*= 0.04). Our conclusions are in accordance with data published by Zee et al. [26] which report a significant association between telomere-associated genes, such as *TERT*, with myocardial infarction risk in the women’s genome health prospective study. Zee et al. [26] examined four SNPs at the *TERC* locus, different from the ones examined here, without finding any significantly associated CHD risk. However, in a single SNP analysis, one of those SNPs, rs10936599 which is in strong LD with rs12696304 (r^2^=0.91 in the HapMap database using the CEU population) was associated with CHD with a hazard ratio of 0.94 [95% CI: 0.85 -1.04] which is comparable to the OR we found for the rs12696304 SNP (OR: 0.90; 95% CI: 0.80 -1.02). However, it is difficult to compare OR and HR. In addition, Matsubara et al. [28] showed that the *TERT* variant rs2735940 was significantly associated with coronary artery disease in a Japanese case-control study. In a large longitudinal study, LTL was predictive of myocardial infarct and death [29], which strengthen the fact that SNPs modulating LTL may have a direct effect on CHD prevalence. 

Specific investigations for the function of the telomerase components on general health have been carried out using knock-out mice models. Terc-/- mice displayed reduced cancer incidence as well as premature aging [30] and third generation Terc-/- mice (G3 Terc-/- mice) showed severely impaired left ventricular function, increased myocyte size and decreased angiogenic potential [31]. Also, G3 *Tert* -/- mice exhibited tissue atrophy, particularly in highly proliferative organs [32] whereas increased *Tert* expression in a cardiac-specific transgenic mouse model reversed the aging phenotype of the heart such as telomere shortening, cell cycle exit in cardiac muscle and cardiac myocyte apoptosis [33]. Overall, functional studies in animal models indicate putative causal effects of telomere and telomerase deficiency on cardiac health, and reinforce our finding that *TERC* variants could confer susceptibility to MI.

With [34], our study is one of the first studies to test the effect of *OBFC1* variants on CHD risk. We found a significant OR of 0.77 for haplotype GC which carries both rare alleles. Of interest, in the Framingham Heart Study, Vasan et al.[35] showed that the *OBFC1* locu*s* is associated with brachial-artery basal blood flow, which is a measure of vascular endothelium function. We showed that the rare allele of the *OBFC1* rs10786775 SNP was also associated with longer LTL and higher expression of *OBFC1* in adventitial aortic tissue. Of note, in the Ensembl database, rs10786775 is located in exon 7 of the gene and induces a functional change, Ser248Cys, in the C-terminal part of the OBFC1 protein [36]. 

The OB Fold-containing Protein 1 (OBFC1) is involved in telomere elongation [37]. Even though the effect of the human OBFC1 on telomeres has not been investigated, the protein is known to protect telomeres [38]. Longer telomeres and activated telomerase would slow down myocyte senescence and promote heart tissue regeneration after injury [39–41]. 

Our data supports the association of *TERC* haplotype with a decreased OR for T2D. In line with our results, Terc-/- mice presented a distinct decrease of average TL in pancreatic islets, impaired glucose tolerance, as well as impaired insulin secretion [42].

Some limitations to our study design need to be considered. Regarding the association between SNPs and LTL, the standard error for LTL measured by quantitative PCR in our study was larger than the standard error for LTL measured by TRF in Mangino et al. [17] and used for the power calculation to estimate the SNP effect size on LTL. This is in accordance with publications comparing both techniques, and our standard error was not larger than that published by other authors measuring LTL by PCR [43,44], however, it is likely that, at least for some of the SNPs, the threshold of power was not reached. To increase the study power, five different studies were meta-analysed. However, the sample was not homogenous; the SB-FH study included FH patients with higher CHD risk and the EARSII study included young subjects at risk of CHD (due to paternal history) and controls. Observing similar results in different population analysed separately would reinforce the association, if any. Moreover, the *TERT* locus was excluded from our initial selection of SNPs, as a result of contradictory data reporting its association with LTL [21-23]. However, it would have been interesting to assess its direct effect on CHD risk. The analyses were performed on cross-sectional data and causality should not be extrapolated, in particular association between SNP and LTL in prevalent CHD or T2D cases may be confounded by the well-known association between short LTL and both CHD and T2D diseases.

The effect of *TERC* and *OBFC1* haplotypes on CHD OR was unchanged after adjustment for LTL. The most likely explanation would be that the SNP effect on LTL was not robust enough in our study (due to large standard error). Also, measuring LTL at a given time point may not be sufficient to estimate the lifetime effect of the SNP on TL and repeated measures and/or measure of the variation in LTL will be needed. 

Despite the SNP modest effect on LTL, an effect was detected for *TERC* and *OBFC1*genes on CHD and T2D OR in the case-control studies presented here. With our study sample size, only the *TERC* haplotype association with T2D (P= 0.007) was maintained after correction for multiple testing. The other associations (including *TERC* and *OBFC1* haplotypes with CHD) were not robust after adjustment for multiple testing. As discussed, small sample size is a major weakness of our study and larger samples would be needed to reach significance after adjustment for multiple testing. Overall, the associations between SNPs associated with LTL and CHD described here are consolidated by very recent publication [34] showing that “although individually the lead SNPs at each of the telomere length–associated loci were not significantly associated with risk of CAD (probably at least in part reflecting their weak individual effects on LTL and low power), in a combined analysis, alleles associated with shorter LTL were associated with a significantly higher risk of CAD.” Replication in large studies and analysis with prospective design would be needed to establish with certainty whether *TERC* and *OBFC1* genes variants have an effect on CHD and T2D risk, via their involvement in TL maintenance. Further research is also required to investigate whether *TERC* and *OBFC1* genes have a direct effect on cardiac health and glucose metabolism, and to determine the underlying mechanism. If this is confirmed, our results may suggest a novel alternative therapy for both heart disease and type 2 diabetes [45].

## Patients and Methods

### Study samples

All studies had been designed and carried out according to the Helsinki declaration and received UCL (university college London) ethics committee approval. All patients gave written informed consent. Details of the studies recruitment and characteristics have been previously published.

#### Hypercoagulability and Impaired Fibrinolytic function MECHanisms (HIFMECH) -case/control study

The HIFMECH study compared 598 male survivors of a first MI aged <60 years (excluding patients with FH and insulin dependent diabetes mellitus) to 653 population-based individuals of the same age and from the same part of Europe [46].

#### Coronary Artery Bypass Graft (CABG)-cases sample

The CABG sample consisted of 439 patients who had an elective first-time coronary artery bypass graft surgery at the Middlesex Hospital, London, UK, between October 1999 and September 2000. Subjects with complications, such as other surgical procedures, pre-existing inflammatory state or unstable coronary artery disease, confounding infective post-operative complications or circulatory failure requiring inotropic support were excluded[47].

#### Simon Broome Familial Hypercholesterolemia study (SB-FH) –Familial hypercholesterolemia cases with nested CHD case/control study

The familial hypercholesterolemia sample (FH) consisted of 410 definite FH adult patients (47.70% women) recruited from the Simon Broome (SB) Familial Hyperlipidaemia Register for FH. Definite FH was defined as total cholesterol concentration >7.5 mmol/l, or LDL-cholesterol concentration>4.9 mmol/l, plus tendon xanthomas in the patient or a first- or second-degree relative [48]. At recruitment, 49.3 % of men and 27.6 % of women had documented CHD with a mean age of onset 43.1 and 46.5 years, respectively [49].

#### University College London Diabetes and Cardiovascular Disease Study (UDACS) -Diabetics cases with nested CHD case/control study

The UDACS study is a cross-sectional sample of diabetic patients selected according to the World Health Organization criteria [50,51]. In the present analysis, a homogeneous sample of 569Caucasian T2D patients was selected. The patients were recruited in London, similarly to the HIFMECH UK controls. The presence of CHD was recorded if the patient had positive coronary angiography/angioplasty, coronary artery bypass, cardiac thallium scan, myocardial infarction, or symptomatic/treated angina. Any individual with a negative investigation or recorded as asymptomatic, was categorized as "no CHD".

#### European Atherosclerosis Research Study II (EARSII) -offspring case/control study

The EARSII sample was recruited in 1993 and consisted of 407 “cases”, male students aged 18 to 28 years whose fathers had a proven MI before the age of 55, compared to 415 age-matched male controls [52]. The students were recruited from 14 Universities throughout 11 European countries. 

### Leukocyte Telomere length measurement

Leukocyte DNA was extracted using the salting-out protocol. LTL was measured with a quantitative polymerase chain reaction (PCR)-based method described by Cawthon [53]. Details of the method were previously described [25]. Briefly, the relative telomere length was calculated as the ratio of telomere repeats to single-copy gene (SCG) copies (T/S ratio). For each sample the quantity of telomere repeats and the quantity of SCG copies were determined in comparison to a reference sample in a telomere and a SCG quantitative PCR, respectively. The reference sample was included in all plates allowing plate standardization. In order to test the reproducibility of the method, 10 randomly chosen samples were run in duplicates on two consecutive days. There was a significant correlation between the measurements obtained on the two different days in linear regression analysis (R2=0.79, p=0.001).The correlation of the lengths' ranking as measured on the two different days was significant (Spearman coefficient=0.82, p=0.004). The coefficient of variation of the T/S ratios in the repeated measurements of the same sample was 5.6%. Using the linear regression line between measures obtained by both the PCR-based method and the conventional terminal restriction fragment (TRF) analysis for the same set of 32 samples, the corresponding telomere length in base pairs (bp) was calculated from the T/S ratio measured in each subject. Measurements in the different studies were performed serially without any changes either in the protocol (including the reference sample) or in the analysis. Results regarding comparison of LTL between CHD cases vs. controls and T2D cases vs. controls were previously published [9,11,25].

### Genotyping

A Pubmed search for ‘genome-wide studies’, which identified SNPs and genes associated with LTL, was performed (Table 2).For the replicated *TERC* locus, two published SNPs were selected. For *OBFC1*, technical difficulties were encountered with rs4387287 genotyping. Thus, the SNP was replaced with rs10786775 and rs11591710 SNPs, both SNPs in high LD with rs4387287 in the HapMap database (r2 rs10786775/rs4387287 was 0.57 and r2 rs11591710/rs4387287 was 0.79 considering the CEU population). 

Additionally, we used our own unpublished genome-wide association data, GWA-AETL (genome-wide association study for aortic endothelial cells telomere length), to supplement the candidate gene list. The GWA-AETL data resulted from a screening performed on 149 human aortic endothelial cell (HAEC) cultures, at an early passage, obtained from healthy donors using the Affymetrix Genome wide Human SNP Array 6.0 assay [54]. The GWA-AETL aimed to identify tissue specific genes involved in TL regulation. In the context of the present article, we used the GWA-AETL data to detect additional SNPs in candidate regionsin order to reinforce the association with TL in diverse tissues, including cardiac cells. Subsequent to the analysis of the GWA data, a list, called AETL, of all SNPs displaying an association with aortic TL (p<0.05) was available. The list included 37978 SNPs categorized by their rs number as well as the gene(s) to which the SNP was more proximal to. Following search by ‘rs’ number, none of the SNPs listed in Table 1 were present in the AETL list. A search throughout the AETL list was performed by gene names. With regard to the 3q26 locus, *TERC* was not found in the AETL list, thus search was extended to *ARPM1, MYNN, LRRC34, LRRIQ4* and *LRRC31*genes [18]. The rs10936601 SNP in the *LRRC34* intron displayed a weak association with TL in aortic cells (β= 0.073, *P* = 0.034).

Genotyping of the seven selected SNPs was carried out by TaqMan technology (Applied Biosystems) and allele calling was generated on an ABI Prism 7900HT (Applied Biosystems) with Sequence Detection Systems 2.1 software. Linkage disequilibrium between the SNPs was obtained using the HapMap database for CEU population and is provided here. 

### Gene expression

The association of rs16847897 (*TERC*) and rs10786775 (*OBFC1*) with gene expression level was examined in the intima-medial and adventitial aorta as previously described in Folkersen et al. [55]. Briefly, the gene expression was measured with Affymetrix Exon 1.0 ST arrays prepared with RMA pre-processing with log_2_-transformation, using the *extended* set of meta probe sets. The genotyping was performed using Illumina 610w Quad chips. The SNPs were not present on the array, so imputation was performed using the Mach 1.0 algorithm [56] together with the 1000 genomes 2010-08 EUR population reference. The rs16847897 and rs10786775 were imputed at acceptable quality (Rsq0.78 and 0.47, respectively). 

### Statistical analysis

Power calculations were performed based on published data by Mangino et al. [17] prior to the present study. In this report, the effect of one allele of the rs2162440 SNP (18q12.2) on LTL was -0.106 (SE 0.022). With our sample size of more than 2300, we would have >99% power to detect an equivalent difference of 110bp per minor allele at the 5% significance level based on a minor allele frequency of 0.21. The minimum difference our study is powered to detect would be 69 base pairs which would be detectable with 80% power. All analyses were limited to Caucasian subjects and data were normally distributed, for some after appropriate transformation (log or square root). In particular, LTL and gene expression was log-transformed. Results are presented as mean or geometric mean plus standard deviation (SD). *P* values were obtained from a two-sample t-test. For each SNP, Hardy-Weinberg equilibrium was assessed using a chi-squared test. For haplotype analysis, SNPs were ranked according to their order in the *ensembl genome browser* database [36]. Haplotypes were designated using the convention of “1” for the common and “2” for the rare allele. Haplotypes were inferred using the *Thesias* program (www.genecanvas.org). LTL was adjusted for age, gender, centre, and exercise (only for HIFMECH) where appropriate using multiple regressions. The β coefficient and standard error (SE) were obtained from regression models with the assumption of an additive effect. The overall effect (β and 95% CI) for the combined studies was obtained by fixed effect meta-analysis using the inverse variance method. The analysis was performed using the metan command in Stata (StataCorp, Texas). In the present work, the composite end point of prevalent total CHD (non-fatal MI, coronary revascularization) events was considered. Data from the offspring study EARSII was included in the meta-analysis, despite the fact that the study design was different (EARSII cases involved offspring of whom the fathers suffered an acute MI before the age of 55 years). 

The odds ratio (OR) and 95% confidence interval (CI) were calculated for all studies using logistic regression. For the HIFMECH study, conditional logistic regression was used to account for individual case-to-control matching. An overall effect (OR (95% CI)) was obtained using fixed effect meta-analysis. P values<0.05 were considered in first instance while a lowest threshold for significance at 0.007 (0.05/7 with 7 been the number of SNPs tested) was defined to account for multiple testing.

Regarding the gene expression data, association tests were carried out using linear additive models of 0-1-2 encoded genotype and gene expression and *R 2*.*14*.*1*software package.

## Supporting Information

File S1 **Supporting Information**.
Table S1. Minor allele frequency (and 95% confidence interval) within each study. Table S2. Test for association of the SNPs with leukocyte telomere length in each study. Figure S1. Forest plot of the effect of TERC haplotype on CHD risk, unadjusted for LTL.
(DOCX)
