# Supplementary material for: Association of TERC and OBFC1 Haplotypes with Mean Leukocyte Telomere Length and Risk for Coronary Heart Disease
Source: PLoS One. 2013 Dec 12;8(12):e83122. doi: 10.1371/journal.pone.0083122 (PMC3861448; doi:10.1371/journal.pone.0083122)
Supplement: File S1 — Supporting Information. Table S1. Minor allele frequency (and 95% confidence interval) within each study. Table S2. Test for association of the SNPs with leukocyte telomere length in each study. Figure S1. Forest plot of the effect of TERC haplotype on CHD risk, unadjusted for LTL. (DOCX) [file pone.0083122.s001.docx]

**Table S1. Minor allele frequency (and 95% confidence interval) within each study**

| SNP | UDACS  - CHD | UDACS  + CHD | HIFMECH  controls | HIFMECH  cases | CABG | SB-FH  - CHD | SB-FH  + CHD | EARS  controls | | EARS  “MI” cases | | |
| --- | --- | --- | --- | --- | --- | --- | --- | --- | --- | --- | --- | --- |
| rs2630578  (*BICD1)* | 0.174  (0.148-0.201) | 0.153  (0.113-0.201) | 0.163  (0.142-0.186) | 0.148  (0.127-0.171) | 0.161  (0.132-0.193) | 0.173  (0.139-0.212) | 0.171  (0.130-0.220) | NA | | NA |  |  |
| rs2162440  (18q12.2) | 0.198  (0.171-0.227) | 0.246  (0.196-0.302) | 0.199  (0.175-0.223) | 0.198  (0.174-0.224) | 0.174  (0.142-0.209) | 0.227  (0.189-0.268) | 0.229  (0.182-0.282) | NA |  | NA |  |  |
| rs16847897  (*TERC)* | 0.274  (0.244-0.306) | 0.248  (0.199-0.303) | 0.304  (0.277-0.331) | 0.300  (0.272-0.329) | 0.295  (0.260-0.333) | 0.249  (0.210-0.291) | 0.286  (0.235-0.342) | 0.282  (0.249-0.316) | | 0.270  (0.238-0.305) | |  |
| rs12696304  (*TERC)* | 0.284  (0.254-0.316) | 0.284  (0.232-0.340) | 0.312  (0.285-0.340) | 0.286  (0.258-0.314) | 0.281  (0.246-0.317) | 0.257  (0.217-0.299) | 0.266  (0.216-0.320) | 0.261  (0.230-0.294) | | 0.251  (0.220-0.285) | |  |
| rs10936601  (*TERC)* | 0.278  (0.247-0.309) | 0.290  (0.237-0.347) | 0.309  (0.282-0.337) | 0.270  (0.243-0.299) | 0.281  (0.246-0.317) | 0.260  (0.220-0.303) | 0.255  (0.207-0.308) | 0.257  (0.225-0.291) | | 0.248  (0.216-0.281) | |  |
| rs10786775  (*OBFC1)* | 0.108  (0.088-0.131) | 0.071  (0.044-0.108) | 0.097  (0.080-0.116) | 0.089  (0.072-0.108) | 0.087  (0.066-0.112) | 0.109  (0.081-0.141) | 0.093  (0.062-0.133) | NA | | NA |  |  |
| rs11591710  (*OBFC1)* | 0.143  (0.120-0.169) | 0.122  (0.086-0.167) | 0.145  (0.125-0.168) | 0.138  0.117-0.160) | 0.118  (0.094-0.145) | 0.141  (0.110-0.176) | 0.115  (0.080-0.157) | NA | | NA |  |  |

**Table S2. Test for association of the SNPs with leukocyte telomere length in each study**

|  | UDACS  - CHD | | UDACS  + CHD | | HIFMECH  controls | | HIFMECH  cases | | CABG | | SB-FH  - CHD | | SB-FH  + CHD | | EARS  controls | | EARS  “MI” cases | | |
| --- | --- | --- | --- | --- | --- | --- | --- | --- | --- | --- | --- | --- | --- | --- | --- | --- | --- | --- | --- |
| SNP | β (SE) | *P* | β (SE) | *P* | β (SE) | *P* | β (SE) | *P* | β (SE) | *P* | β (SE) | *P* | β (SE) | *P* | β (SE) | *P* | β (SE) | *P* |  |
| rs2630578  (*BICD1)* | -0.006 (0.020) | 0.78 | 0.032 (0.036) | 0.39 | 0.045  (0.027) | *0.09* | -0.0004  (0.024) | 0.99 | 0.0165  (0.041) | 0.68 | -0.038 (0.040) | 0.35 | 0.019  (0.050) | 0.71 | NA |  | NA |  |  |
| rs2162440  (18q12.2) | 0.006 (0.018) | 0.76 | -0.007 (0.030) | 0.83 | 0.020  (0.026) | 0.44 | -0.016  (0.022) | 0.46 | -0.014  (0.041) | 0.72 | -0.059 (0.037) | *0.11* | -0.022 (0.048) | 0.65 | NA |  | NA |  |  |
| rs16847897  (*TERC)* | 0.006 (0.016) | 0.72 | -0.030 (0.012) | 0.30 | -0.020  (0.022) | 0.37 | 0.012 (0.019) | 0.54 | 0.024  (0.032) | 0.46 | 0.055  (0.037) | 0.13 | -0.020  (0.043) | 0.65 | 0.050  (0.052) | 0.33 | -0.025  (0.054) | 0.65 |  |
| rs12696304  (*TERC)* | 0.011 (0.016) | 0.48 | -0.049 (0.030) | *0.10* | -0.023 (0.021) | 0.27 | 0.007 (0.018) | 0.70 | 0.019  (0.031) | 0.53 | 0.004  (0.036) | 0.90 | -0.080  (0.048) | *0.09* | 0.053  (0.051) | 0.31 | -0.028  (0.055) | 0.62 |  |
| rs10936601  (*TERC)* | 0.009 (0.016) | 0.58 | -0.054 (0.030) | *0.07* | -0.018  (0.021) | 0.39 | 0.014 (0.019) | 0.45 | 0.025  (0.031) | 0.42 | -0.009  (0.037) | 0.81 | -0.071  (0.047) | *0.13* | 0.060  (0.053) | 0.26 | -0.047  (0.056) | 0.40 |  |
| rs10786775  (*OBFC1)* | 0.018 (0.023) | 0.43 | 0.054 (0.050) | 0.28 | 0.036  (0.035) | 0.31 | 0.030  (0.030) | 0.31 | -0.008  (0.049) | 0.87 | 0.093 (0.051) | *0.07* | -0.09 (0.069) | 0.90 | NA |  | NA |  |  |
| rs11591710  (*OBFC1)* | 0.004 (0.020) | 0.83 | 0.037 (0.041) | 0.36 | 0.020  (0.030) | 0.50 | -0.004  (0.025) | 0.88 | -0.047  (0.045) | 0.31 | 0.113 (0.043) | 0.01 | -0.089 (0.070) | 0.20 | NA |  | NA |  |  |

NA, non available; SE, standard error

Additive model fixed effect

Linear regression adjusted for age, gender, center and physical activity as appropriate.

**Supplement figure 1. Forest plot of the effect of *TERC* haplotype on CHD risk, unadjusted for LTL**
